# Simultaneous Bilateral Femoral and Tibial Lengthening in Achondroplasia

**DOI:** 10.3390/children8090749

**Published:** 2021-08-30

**Authors:** Lior Shabtai, Julio J. Jauregui, John E. Herzenberg, Martin G. Gesheff, Shawn C. Standard, Philip K. McClure

**Affiliations:** 1 Zucker School of Medicine at Hofstra/Northwell, 500 Hofstra Blvd, Hempstead, NY 11549, USA; lish3@hotmail.com; 2 Department of Orthopaedics, University of Maryland Medical Center, 110 S. Paca Street, 6th Floor, Suite 300, Baltimore, MD 21201, USA; juljau@gmail.com; 3 International Center for Limb Lengthening, Rubin Institute for Advanced Orthopedics, Sinai Hospital of Baltimore, 2401 W. Belvedere Ave, Baltimore, MD 21215, USA; jherzenb@lifebridgehealth.org (J.E.H.); mgesheff@lifebridgehealth.org (M.G.G.); sstandar@lifebridgehealth.org (S.C.S.)

**Keywords:** bone lengthening, achondroplasia, external fixation, distraction osteogenesis

## Abstract

Previous studies on lengthening for achondroplasia have reported bilateral extensive femoral lengthening followed by bilateral extensive tibial lengthening. To decrease trauma on soft tissues and joints, we propose bilateral simultaneous moderate femoral lengthening and moderate tibial lengthening followed by a similar repeat lengthening a few years later. Fifty patients with achondroplasia underwent 65 simultaneous bilateral femoral and tibial lengthening procedures. Segment lengthening amount and adverse events were obtained from medical records. Mean follow-up after bone healing was 35.6 months. Mean tibial lengthening was 52 mm; mean femoral lengthening was 72 mm. Average healing index was 1.4 months/cm for the tibia and 1 month/cm for the femur. Mean duration of treatment with external fixation was 6.7 months (range, 4.4–10.5 months). Thirty-eight (76%) of 50 patients experienced one or more adverse events during lengthening. We observed 78 adverse events, 35 (45%) of which required additional surgical procedures. All resolved by the end of treatment. Mechanical axis deviation improved from a mean of 15 mm medially to 8 mm medially. Simultaneous lengthening of four segments in patients with achondroplasia is a feasible strategy. Compared with isolated femoral or tibial lengthening, distributing the lengthening between the femur and tibia decreases total external fixator time.

## 1. Introduction

Achondroplasia is the most common dwarfing disorder affecting humans, with an incidence of one in 20,000 to 30,000 live births [1]. The underlying genetic difference is a mutation in the FGFR3 gene, leading to abnormal endochondral ossification and rhizomelic short stature (average total height between 130 and 140 cm) [2]. Limb lengthening in patients with achondroplasia is controversial. Supporters claim that there are purposeful and psychosocial benefits of achieving functional height (i.e., greater than 150 cm) [3,4,5]. A study by Kim et al. [5] showed that patients with achondroplasia tolerate a greater amount of lengthening with fewer complications compared with patients of other etiologies. Opponents claim that achondroplasia is a normal variant and that limb lengthening does not address any medical problem and may put patients at risk for disability secondary to complications [6]. In addition, one lengthening procedure is usually not adequate and therefore serial lengthening procedures are needed to achieve a satisfactory and desired height. While multiple trials are underway with experimental agents for treatment of some features of achondroplasia, it is likely that a role for surgical limb lengthening in patients with functional deficits will persist in the near future.

**Figure 1:**
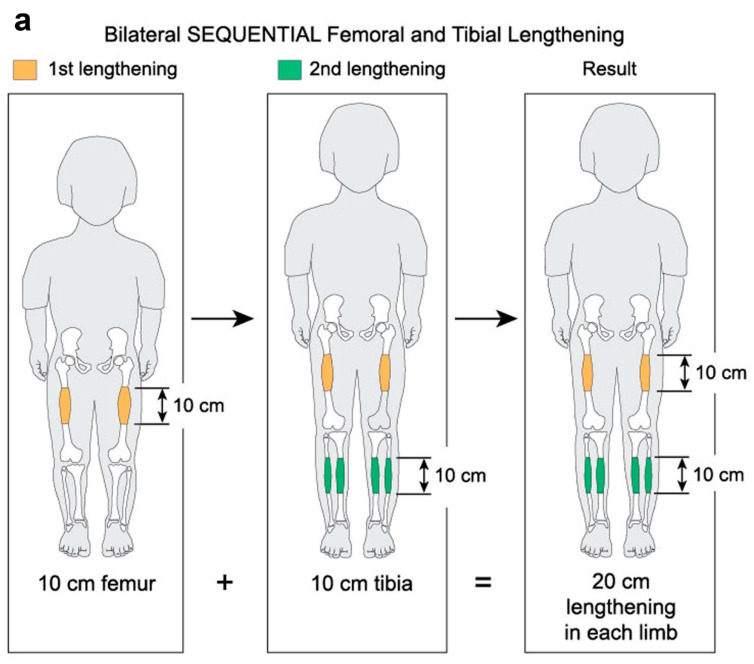

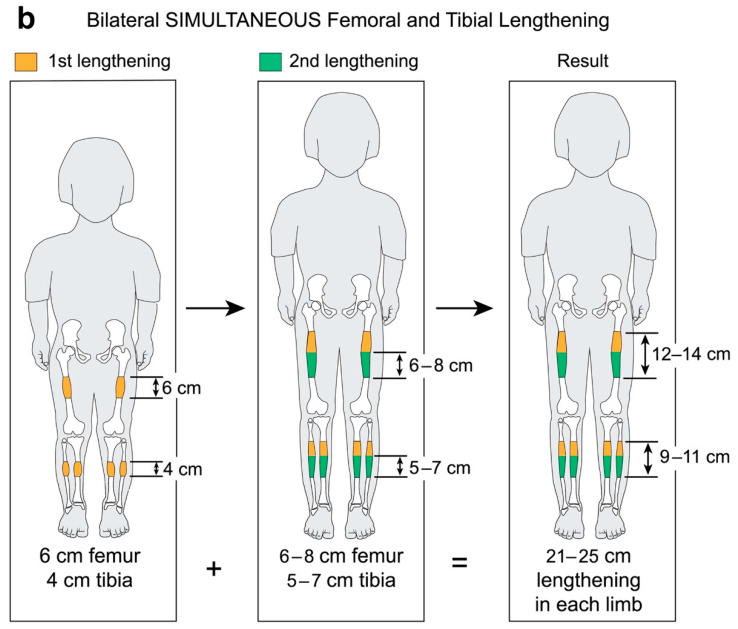
(**a**) Sequential extensive lengthening. (**b**) Moderate simultaneous bilateral femoral tibial lengthening (SBFTL). Both the extensive and the moderate strategies result in the same 20 cm of total lengthening (used with permission).

Limb lengthening with external fixators for achondroplasia has been reported by many groups [7,8,9,10,11]. Previous studies have reported bilateral extensive (~10 cm) femoral lengthening followed two years later by bilateral extensive (~10 cm) tibial lengthening. Such large individual lengthening can be very stressful on the limb (Figure 1a). A large (10 cm) lengthening of the tibia, for example, puts a large amount of stretching on the peroneal nerve. A previous article from this institute on double level tibia (10 cm) showed a very high peroneal nerve palsy rate [12].

In an effort to decrease the trauma to soft tissues and joints, we propose bilateral simultaneous moderate (5–7 cm) femoral lengthening and moderate (4–5 cm) tibial lengthening (Figure 1b). A few years after the initial lengthening, a second lengthening is performed. The specific length of time depends upon the desires of the family and child. The second lengthening consists of bilateral femoral lengthening (6–8 cm) and simultaneous bilateral tibial lengthening (4–6 cm). While extensive lengthening results in 20 cm of total lengthening, moderate lengthening may result in 21 to 25 cm of total lengthening (Figure 1b). We term this approach simultaneous bilateral femoral tibial lengthening (SBFTL).

We propose to (1) assess the outcomes of simultaneous moderate four-segment lengthening; (2) to compare this method with consecutive lengthening as presented in the literature; and (3) to assess whether secondary lengthening procedures and/or the magnitude of these lengthening procedures are associated with a higher incidence of adverse events.

## 2. Materials and Methods

The study was conducted according to the guidelines of the Declaration of Helsinki, and the LifeBridge Health Institutional Review Board determined that this study was exempt from full review (protocol #1587, 12/2/2009). Informed consent was not required, as it was a retrospective review of medical records of all patients with achondroplasia who underwent SBFTL. A total of 64 achondroplasia patients (256 bone segments) underwent SBFTL between January 2002 and December 2012 at our institution. The inclusion criteria were simultaneous four-segment lengthening using femoral and tibial external fixation. A minimum of 12 months of follow-up after external fixation removal was also required to be included in the study.

**Figure 2:**
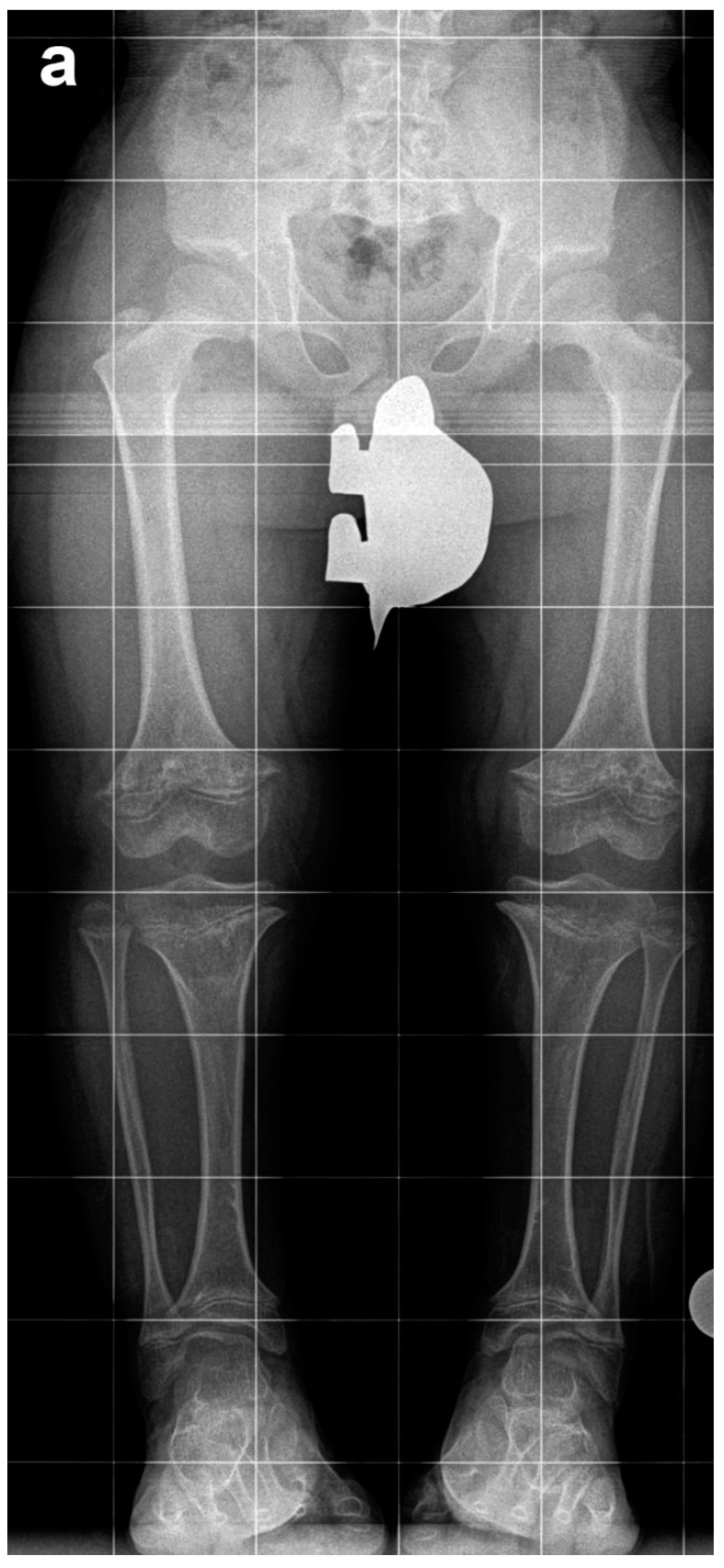

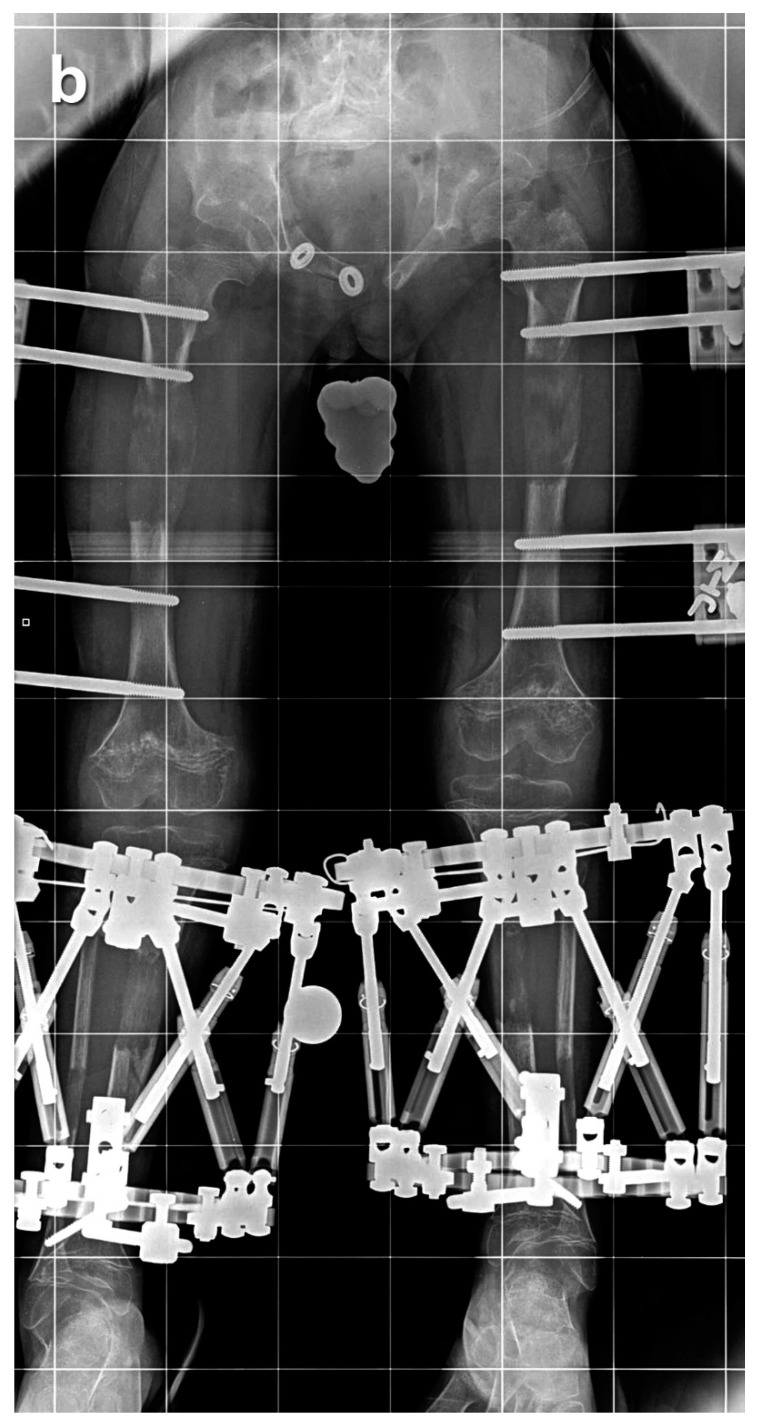

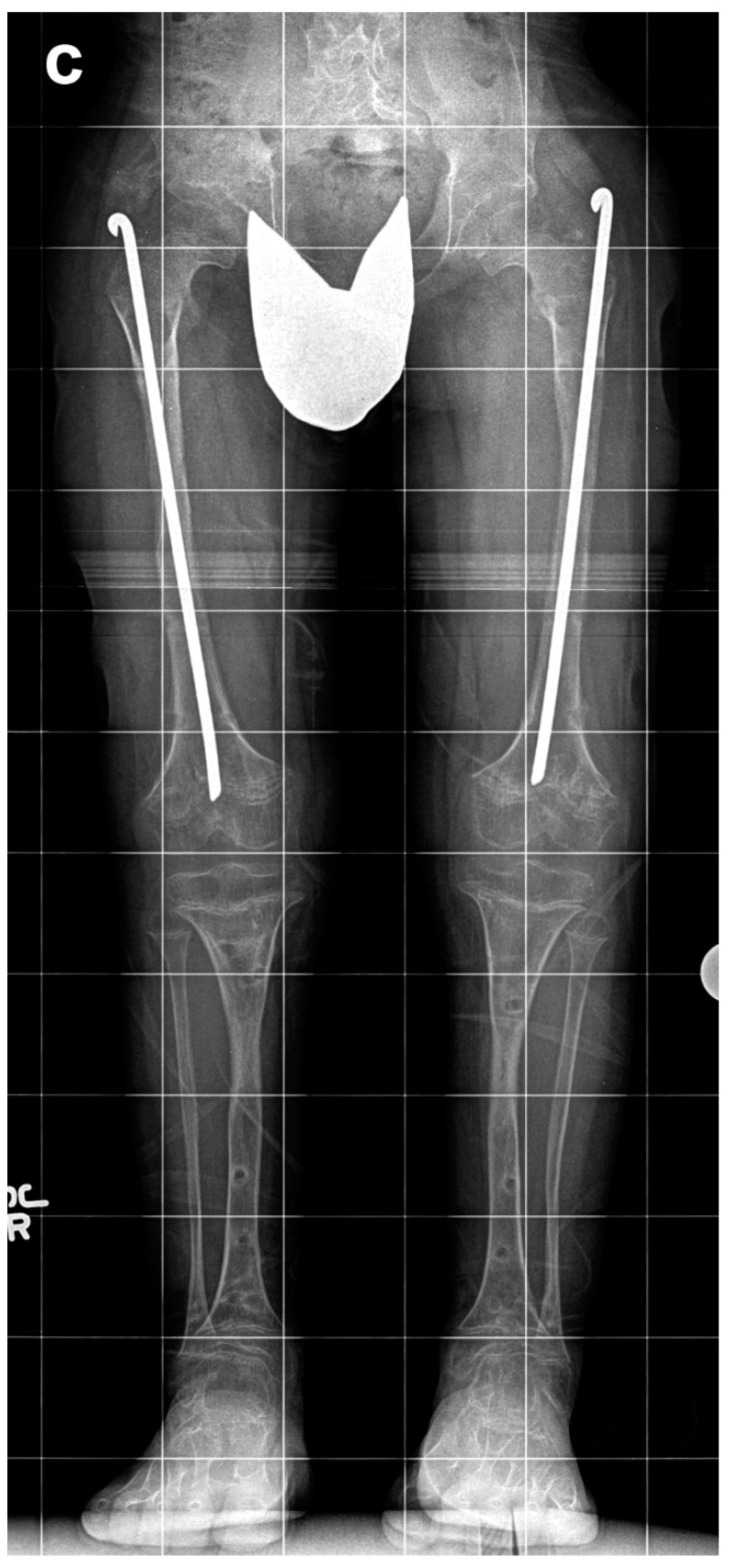

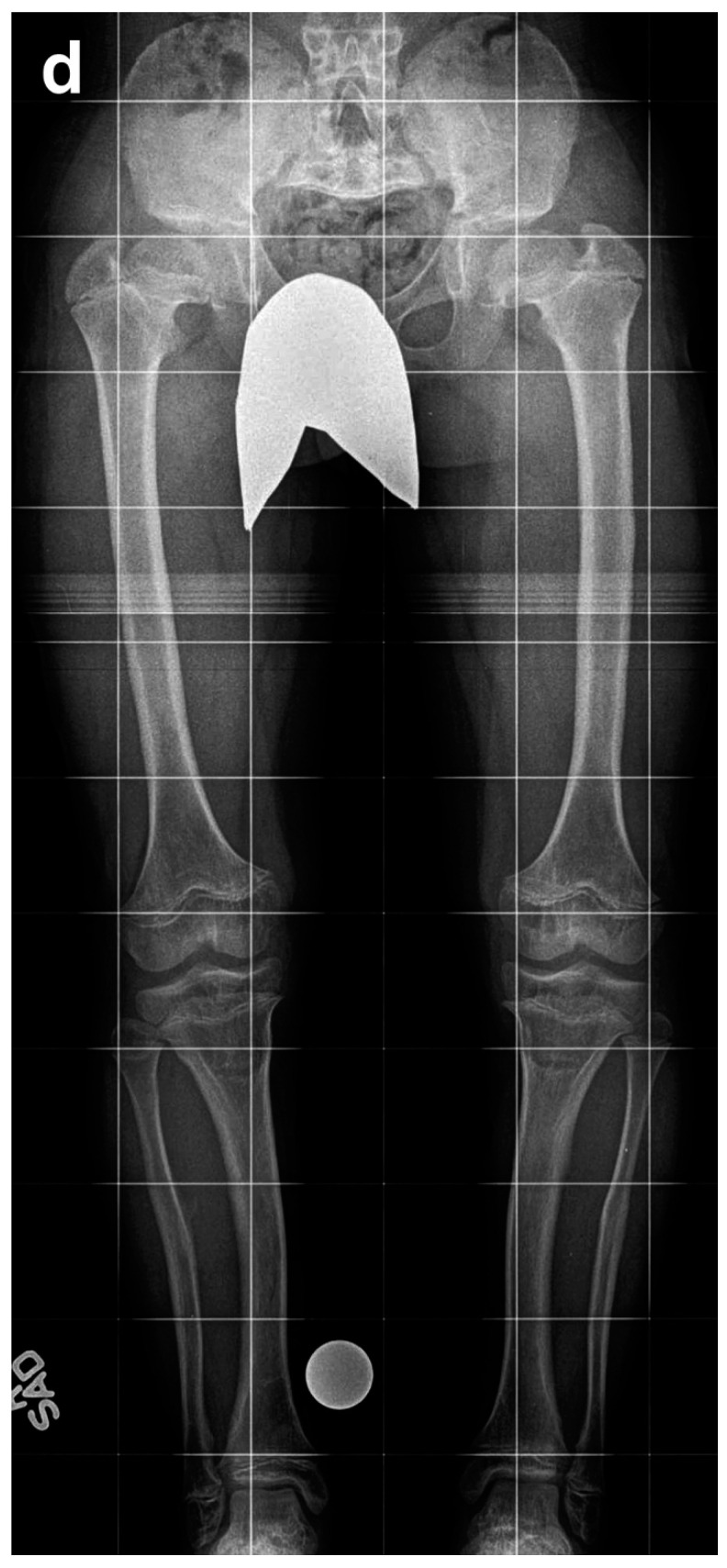

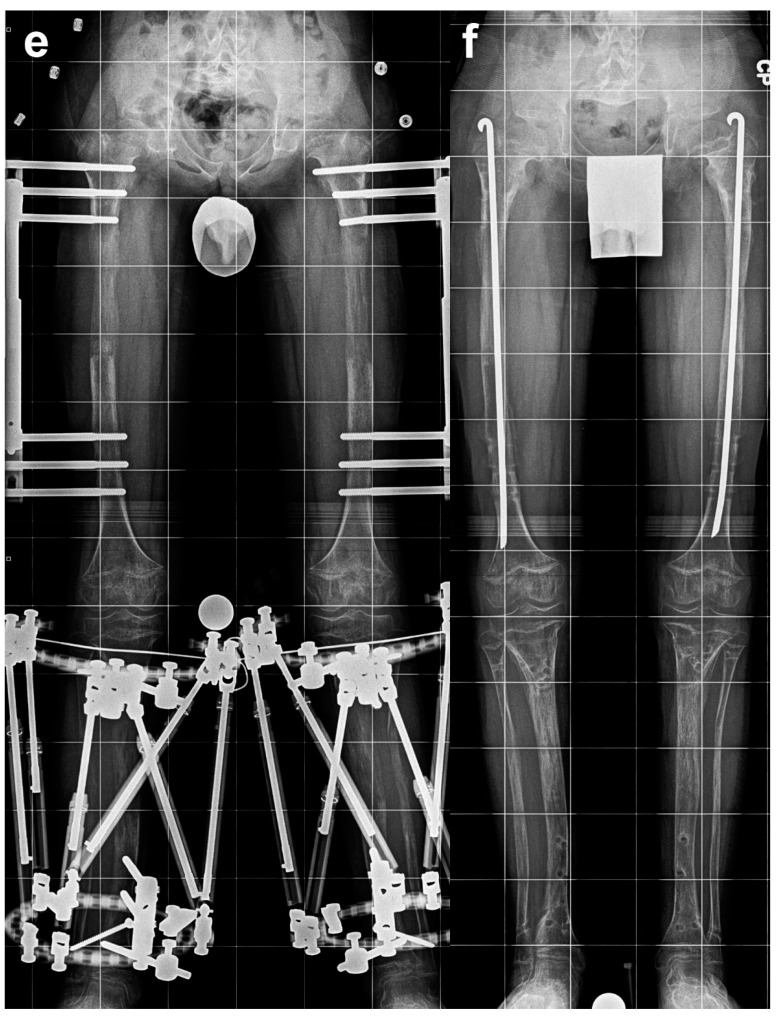
Eight-year-old boy who underwent two simultaneous bilateral femoral tibial lengthening (SBFTL) procedures. A Taylor Spatial Frame (Smith & Nephew, London, UK) was used for the tibia, and an Orthofix LRS (Lewisville, TX) was used for the femur. (**a**) Prior to first limb lengthening. (**b**) During first SBFTL. (**c**) Status after 10-cm lengthening. (**d**) Prior to second limb lengthening. (**e**) During second SBFTL. (**f**) Status after additional 12.5-cm lengthening (used with permission).

Of these 64 patients, nine patients were excluded as they underwent SBFTL using other methods of fixation, such as internal femoral nail or a combination of internal fixation for the femur and external fixation for the tibia. An additional five patients did not have at least 12 months of follow-up after external fixation removal and were excluded from this analysis. Fifty patients (29 males, 21 females, 200 bone segments) with achondroplasia met our inclusion criteria and underwent a total of 65 lengthening procedures (260 bone segments underwent lengthening). We defined a “procedure” as SBFTL (Figure 2).

Demographic data, duration of follow-up, lengthening parameters, and adverse events were obtained from the medical records. Amount of lengthening of each bone, duration of treatment with external fixation, and healing index were recorded. Healing index was defined as time (months) from the index surgery until external fixation removal, divided by amount of lengthening achieved (centimeters). Mechanical axial deviation was measured before and after lengthening. Range of motion of the hip, knee, and ankle was measured before surgery, after surgery, and at final follow-up. Adverse events during the lengthening procedures were classified into problems, obstacles, and complications [13]. Problems required no surgical intervention to resolve, while obstacles required surgical intervention. Interoperative injuries, adverse events that did not resolve before the end of treatment, and adverse events that interfered with the goals of treatment were classified as true complications.

## 3. Results

Fifty patients (29 boys, 21 girls) with achondroplasia underwent a total of 65 SBFTL procedures. Thirty-six patients underwent one SBFTL, 13 patients underwent two SBFTL procedures, and one patient underwent three SBFTL procedures. The mean age at the first lengthening was 11.8 years (range, 7.1–29.3 years), while the mean age at the second lengthening was 13.6 years (range, 11.5–17.2 years). The single patient who had three SBFTL procedures underwent the most recent one at age 13.8 years.

The mean follow-up after bone healing was 35.6 months (range, 12–102 months). The mean duration of treatment with external fixation was 6.7 months (range, 4.4–10.5 months). The mean tibial lengthening per SBFTL procedure was 52 mm (range, 25–79 mm), and the mean femoral lengthening per SBFTL procedure was 72 mm (range, 11–105 mm). The average healing index was 1.4 months/cm for the tibia (range, 0.6–2.2 months/cm) and 1.0 month/cm for the femur (range, 0.6–4.6 months/cm). The mechanical axis deviation improved from a mean of 15 mm to 8 mm. Mean hip, knee, and ankle range of motion did not change after treatment.

All patients except one achieved the planned lengthening. Patients were seen every 10 to 14 days, and we would modify up or down from the standard of 4 × ¼ mm per day dependent on the quality and quantity of the regenerate. The minimum length that we accept from each segment is 4 to 5 cm; therefore, if the premature consolidation occurs prior to achieving 4 to 5 cm, we would strongly advise re-osteotomy in order to achieve our minimum desired length. One patient developed nonunion following compartment syndrome during the second lengthening procedure and elected not to complete the lengthening. The patient gained 2.5 cm in the tibia and 1.1 cm in the femur. Therefore, the success rate per lengthening session, defined as the number of lengthening sessions in which the desired length was achieved, was 64 (98.4%) of 65 lengthening sessions.

Thirty-eight (76%) of 50 patients experienced one or more adverse events during one of the SBFTL procedures. In total, 78 adverse events occurred. Forty-three were classified as “problems”, such as pin-tract infection and muscle contractures, which all resolved by oral antibiotic and physiotherapy, respectively. Thirty-three of 78 adverse events were classified as “obstacles” and required additional surgical procedures, and all 33 resolved by the end of treatment. The true rate of obstacles was 0.127 (33/260) per bone segment. The 33 obstacles included ten peroneal nerve entrapments, eight premature consolidations, four fractures after frame removal, two cases of compartment syndrome, three malunions, two fractures prior to frame removal, two scar contractures, one nonunion, and one deep infection. Two complications (nonunion, compartment syndrome) occurred in the same patient, preventing the achievement of the lengthening goal.

The average length of time from the first to the second procedure was 3.5 years (range, 2–5 years). Comparing the rate of complication with a T test between the first and the second lengthening, a statistically significant difference was not found (*p* = 0.68). In addition, there was no statistically significant difference between the healing indexes of the tibia or femur for first time SBFTL compared to second time SBFTL. The healing index in the first lengthening was 1.4 months/cm and 1.0 months/cm for the tibia and the femur, respectively, compared to 1.3 months/cm and 1.2 months/cm, respectively, in the second lengthening (*p* = 0.47).

Concerning postoperative rehabilitation, for all bilateral lengthening patients, we allow limited 4-point gait walking in the house, gradually progressing to full weight-bearing as the bone heals. We also incorporate pool therapy as it permits patients to be semi weight-bearing.

## 4. Discussion

Extensive femoral and tibial lengthenings are stressful events for the limb and for the patient [12]. Various adverse events are associated with extensive limb lengthening, including damage to the surrounding soft tissue, joint dislocations, and muscle contractures. In addition, extensive lengthening may lead to excessive pressure on open growth plates, resulting in physeal growth inhibition [8,9,14]. In this study, we used bilateral simultaneous moderate femoral lengthening and tibial lengthening. Utilizing this method, we observed a lower complication rate than what has been previously reported in the literature for limb lengthening in patients with achondroplasia [7,8,9,10,11].

Our study has three limitations that must be considered. First, this is a retrospective, non-comparative study performed at a single center. There is no direct comparison of extensive versus moderate lengthening. Instead, we compared our results with other outcomes reported in the literature. Second, we did not include a satisfaction questionnaire in this study, which could be important for understanding the patient experience in conjunction with the clinical outcomes. However, Kim et al. [3] demonstrated that limb lengthening improves the quality of life for achondroplastic patients even though it is associated with numerous complications. Third, other methods of limb lengthening are now available, such as intramedullary lengthening nails, which may be associated with fewer complications and increased patient satisfaction. We did not include patients who underwent intramedullary limb lengthening in this study. In a previous study of femoral lengthening, we found a 30% rate of refracture after external fixator removal. Therefore, in any cases where the regenerate bone is less than fully robust, our recommendation is to insert a prophylactic rush rod to prevent bending of the regenerate. This can be especially important in bilateral cases, since the ability to restrict weight-bearing is limited [15].

All lengthening sessions achieved the planned lengthening except for one, resulting in a success rate of 98.4%. The mean tibial lengthening was 5.2 cm, and the mean femoral lengthening was 7.2 cm, which is an average of a 12.4-cm gain per limb in one procedure. Our hypothesis is that by dividing this amount of lengthening between two bone segments instead of extensively lengthening only one segment, the trauma on soft tissues and joints is reduced. This may explain why our complication rate is lower than that reported in the literature [7,8,9,10,11]. These outcomes are similar to a 2014 study of SBFTL published by Kocaoglu et al. [16]. They treated 22 achondroplastic patients (88 bones segments) with SBFTL and demonstrated a lower complication rate and a decrease in time of total external fixation treatment. Our study emphasizes the safety and the efficacy of SBFTL with large numbers (50 patients, 260 bone segments underwent lengthening). In addition, 26% (13/50) of our patients underwent secondary four-segment lengthening without an increased rate of adverse events.

We believe that simultaneous, moderate, four-segment lengthening is safer than consecutive extensive lengthening. We observed adverse events that were classified as obstacles and complications at a rate of 0.135 (35/260) per bone segment, which is lower than what has been reported in other studies [3,4,5,7,11,17,18]. Both Lie et al. [17] and Vaidya et al. [11] presented a complication rate of 0.95 per bone segment in different studies. Kim et al. [5] had 117 complications required surgery in 88 segments with a complication rate of 1.3 (117/80) per bone segment. Regarding the amount of lengthening, Venkatesh et al. [19] studied the relationship between the amount of femoral lengthening and the complication rate in 20 patients with achondroplasia (40 femora). They found that patients who lengthened the femur by more than 50% of its original length had higher rates of complication. We strongly agree with this; therefore, the mean femoral and tibia lengthening amounts per lengthening procedure in this study were relatively moderate, at 7.2 cm and 5.2 cm, respectively. These values are similar to the values presented by Venkatesh et al. [19] for the group, with fewer complications. The true limits of lengthening are the soft-tissue compliance, the joint range of motion, and the quality of the bone [13]. Lengthening between 5 cm and 8 cm has a medium risk of complication, while lengthening beyond 8 cm may result in a higher complication rate. A recent article showed that it can be done with internal fixation as well [20]. A portion of our study’s dataset (from 2002 to 2009) may overlap with the same 7-year portion of this recent article’s dataset.

We did not find a higher rate of adverse events in patients who underwent subsequent lengthening in the same bone. This outcome was demonstrated also by Griffith et al. [21], who reported similar complication rates for both the first and the second lengthening procedures. However, they did find that the healing index time was slower for both the femur and tibia in the second lengthening procedure. In our study, there was no statistically significant difference between the healing index of the first lengthening and the second lengthening (*p* = 0.47). We believe that one of the reasons for the similar outcomes for the first lengthening and the second lengthening is that the average duration of time between the first and the second procedure was 3.5 years (range, 2–5 years). There is a minimum of 2 years between surgeries that can be modified by the family according to social and psychological considerations. Tjernström et al. [22] demonstrated that the time required for bone remodeling after lengthening is at least one year.

In summary, simultaneous bilateral femoral and tibial lengthening in patients with achondroplasia is a feasible procedure. We observed a lower complication rate than what has been reported in the literature for limb lengthening in patients with achondroplasia. Total external fixation time is decreased by dividing the total lengthening goal between the femur and tibia compared with concentrating all the lengthening in only the femur or only the tibia. None were done with internal fixation; all were external fixation. More recently, we have further modified this technique to include intramedullary nails.

## Data Availability

The data presented in this study are available upon request from the corresponding author. The data are not publicly available due to privacy concerns with protected health information.

## Abbreviations

The following abbreviation is used in this manuscript:

SBFTL	simultaneous bilateral femoral tibial lengthening
